# AtSWEET1 negatively regulates plant susceptibility to root-knot nematode disease

**DOI:** 10.3389/fpls.2023.1010348

**Published:** 2023-02-07

**Authors:** Yuan Zhou, Dan Zhao, Yuxi Duan, Lijie Chen, Haiyan Fan, Yuanyuan Wang, Xiaoyu Liu, Li-Qing Chen, Yuanhu Xuan, Xiaofeng Zhu

**Affiliations:** ^1^ Nematology Institute of Northern China, College of Plant Protection, Shenyang Agriculture University, Shenyang, China; ^2^ College of Plant Protection, Jilin Agricultural University, Changchun, China; ^3^ College of Biological Science and Technology, Shenyang Agriculture University, Shenyang, China; ^4^ College of Sciences, Shenyang Agriculture University, Shenyang, China; ^5^ Department of Plant Biology, University of Illinois at Urbana-Champaign, Urbana, IL, United States

**Keywords:** *Arabidopsis thaliana*, giant cells, gene expression, *Meloidogyne incognita*, sugar transporters

## Abstract

The root-knot nematode *Meloidogyne incognita* is a pathogenic pest that causes severe economic loss to agricultural production by forming a parasitic relationship with its hosts. During the development of *M. incognita* in the host plant roots, giant cells are formed as a nutrient sink. However, the roles of sugar transporters during the giant cells gain sugar from the plant cells are needed to improve. Meanwhile, the eventual function of sugars will eventually be exported transporters (SWEETs) in nematode-plant interactions remains unclear. In this study, the expression patterns of *Arabidopsis thaliana SWEETs* were examined by inoculation with *M. incognita* at 3 days post inoculation (dpi) (penetration stage) and 18 dpi (developing stage). We found that few *AtSWEETs* responded sensitively to *M. incognita* inoculation, with the highest induction of *AtSWEET1* (AT1G21460), a glucose transporter gene. Histological analyses indicated that the β-glucuronidase (*GUS*) and green fluorescent protein (*GFP*) signals were observed specifically in the galls of AtSWEET1-GUS and AtSWEET1-GFP transgenic plant roots, suggesting that *AtSWEET1* was induced specifically in the galls. Genetic studies have shown that parasitism of *M. incognita* was significantly affected in *atsweet1* compared to wild-type and complementation plants. In addition, parasitism of *M. incognita* was significantly affected in *atsweet10* but not in *atsweet13* and *atsweet14*, expression of which was induced by inoculation with *M. incognita*. Taken together, these data prove that SWEETs play important roles in plant and nematode interactions.

## 1 Introduction

Plant parasitic nematodes are responsible for over US$157 billion worth of annual crop losses worldwide (Abad et al., 2008). Root-knot nematode (RKN) is the most important one out of the ten most damaging plant parasitic nematodes, causing significant economic losses every year (Jones et al., 2013). RKN has a wide host range, parasitizing vegetables, fruit trees, and ornamental plants and has been spreading worldwide (Triantaphyllou, 1985). *Meloidogyne incognita*, as one of the root-knot nematodes, interacts with the hosts *via* a complex process. Second-stage juveniles (J2s) infiltrate the root at the elongation zone, migrate to the tip, and then turn 180° to enter the vascular cylinder and proceed upwards until they reach the differentiation zone, where they generate numerous giant cells (GCs) (von Mende, 1997; Bird et al., 2009; Perry and Moens, 2011; Escobar et al., 2015; Cabrera et al., 2018). Neighboring cells multiply in the vascular cylinder as GCs mature, and cortical cells hypertrophy, generating a root-knot formation known as a gall. Within a gall, RKNs become sedentary and feed on GCs until the life cycle is complete (Dropkin, 1972; Wyss et al., 1992).

Sijmons first established culture conditions to successfully establish a new model system for studying the infection and development of *M. incognita* in *Arabidopsis thaliana* (Sijmons et al., 1991). The infection of *A. thaliana* with *M. incognita* is a well-suited model system for studying the molecular interactions between nematodes and their hosts (Engler et al., 2016; Singh et al., 2016; Teixeira et al., 2016; Warmerdam et al., 2018; Warmerdam et al., 2020). Teixeira and Warmerdam studied early defense responses against *M. incognita* in *A. thaliana*. Singh discussed the establishment of GCs in *M. incognita* and *A. thaliana* interactions. Engler identified several cell cycle genes driving giant cell development in *A. thaliana* infected with *M. incognita*.

RKN infections are tightly linked to changes in sugar concentration in plants. GCs serve as a nutrient sink for developing nematodes, where the metabolism of carbohydrates and amino acids is highly active (Baldacci-Cresp et al., 2012; Machado et al., 2012; Gautam and Poddar, 2014). The amount of sucrose in root exudates from tomato plants infected with *M. incognita* was double that of healthy root exudates (Wang and Bergeson, 1974). According to sensitive metabolomics methods, galls formed by *M. incognita* in *Medicago truncatula* have enhanced amounts of starch, sucrose, and glucose (Baldacci-Cresp et al., 2012). Coffee and bitter gourd roots infected with *Meloidogyne exigua* and *M. incognita* yielded similar results (Machado et al., 2012; Gautam and Poddar, 2014). *Trehalase 1* (TH1) gene is an enzyme that catalyzes the hydrolysis of trehalose, which was more highly expressed in the female stage of *M. incognita* in *Nicotiana benthamiana* than in eggs or in the parasitic stage (Mani et al., 2020). During the nematode feeding sites establishment, transporters are responsible for sucrose supply (Hofmann et al., 2007). AtSUC2 and AtSUC4, specific sucrose transporters, are activated by *Heterodera schachtii* and mediate the transmembrane transport of sucrose in the syncytia (Juergensen et al., 2003).

Sugars will eventually be exported transporters (SWEETs) are a family of sugar transporters that play a role in phloem loading and pathogen nutrition utilization by mediating the uptake and extrusion of sugars across the cellular membrane (Chen, 2013; Xue et al., 2022). As a novel characteristic sugar transporter group, SWEETs have been identified to bidirectionally transport monosaccharides and polysaccharides and are closely involved in the interaction process between pathogenic microorganisms and host plants (Chen et al., 2010; Li et al., 2013; Chong et al., 2014; Cohn et al., 2014; Hu et al., 2014; Gao et al., 2018). By analyzing all SWEETs in *A. thaliana*, the four prolines in the TM1, TM2, TM5, and TM6 domains were found to bind and transport sugars by stimulating structural rearrangements to generate homologous or heterologous polymers by altering the protein conformation (Tao et al., 2015). Chen et al. (2010) used a high-sensitivity fluorescence resonance energy transfer (FRET) glucose sensor to determine that AtSWEET1 is a glucose uniporter (Chen et al., 2010),and AtSWEET10, AtSWEET13, and AtSWEET14 are sucrose uniporters (Chen et al., 2012). *Arabidopsis AtSWEET1* is strongly expressed in stamen primordia and during the early stages of floral development, according to an *in situ* hybridization study (Wellmer et al., 2006).

*SWEET* genes are involved in the interaction between pathogens and their hosts. It has been reported that *AtSWEET1* expression was highly induced by *Pseudomonas syringae* pv. *tomato* strain DC3000 and *Golovinomyces cichoracearum*, indicating a potential role in pathogen nutrition (Chen et al., 2010). Several *AtSWEET* genes are induced by *Pseudomonas syringae* pv. *tomato* DC3000, *Golovinomyces cichoracearum*, and *Botrytis cinerea* in the model plant *A. thaliana*; all three fungi have potential roles in pathogen nutrition (Chen et al., 2010). When *M. incognita* infects a tomato (*Solanum lycopersicum*), 17 *SlSWEETs* are up-regulated in the host’s leaves and roots, with 10 upregulated in both tissues (Zhao et al., 2018). The expression of eight SWEET genes is altered in soybean plants after inoculation with *Rotylenchulus reinformis* (Redding et al., 2018).

The syncytia are symplasmically isolated from surrounding tissues in Arabidopsis, which indicates that the syncytia obtain nutrients from their surrounding tissues requires protein carriers (Juergensen et al., 2003). Several studies demonstrated that AtSWEETs play a vital role in bridging nutrient communication during different plant-pathogen interactions, which encourages us to investigate the potential role of AtSWEETs in the roots of *A. thaliana* upon infection by RKNs. In this study, we showed several *AtSWEETs* were induced by the infection of RKNs. Among these induced *AtSWEETs*, we conducted further studies for *AtSWEET1* to gain the spacial expression information using histochemical staining of β-glucuronidase (GUS) assay. We also aimed to determine the role of AtSWEET1 expression on RKN invasion, development and gall formation in *A. thaliana*.

## 2 Materials and methods

### 2.1 Plant material and growth conditions

The 4056-bp genomic fragment containing the SWEET1 promoter and coding region was amplified using the primers SWEET1CMF-1 (5’-GGGGACAAGTTTGTACAAAAAAGCAGGCTTACCGCTTGTTCCATTCATTCTGATT-3’) and SWEET1CMR-1 (5’-GGGGACCACTTTGTACAAGAAAGCTGGGTAAACTTGAAGGTCTTGCTTTCCATT-3’) and cloned into the entry vector pDORN221-f1. The pSWEET1:gSWEET1 cassette was switched to the binary vectors pEG-TW1-EYFP and pMDC163 *via* LR reaction to make pSWEET1::gSWEET1-YFP and pSWEET1::gSWEET1-GUS, respectively.

*Arabidopsis thaliana* ecotype Col-0 and mutant genotypes in the Col-0 background, *atsweet1*, AtSWEET1-GUS, AtSWEET1-YFP-w5-2, and AtSWEET1-YFP-S2-2, were cultured in a growth chamber at 22°C under a 12h/12h light/dark cycle. For plant cultivation, sand was passed through a 20-mesh (0.85 mm) sieve, sterilized at 165°C for 2 h, and placed in a clean culture tube with a diameter of 4 cm and a height of 13.5 cm. The sand was moistened with sterile water. *Arabidopsis* seeds (wild-type Col-0 and mutant) were planted directly on the surface of the sand and covered with a transparent film to maintain humidity.

### 2.2 Fresh weight statistics of *atsweet1* and Col-0 roots

*AtSWEET1* is a glucose transporter gene, one of the most important carbohydrates in plants, that plays an important role in Arabidopsis growth and development (Wellmer et al., 2006; Chen et al., 2010). To test the effect of *AtSWEET1* gene mutation on Arabidopsis root growth, *atsweet1* and Col-0 seeds were sown on a sand surface. After 35 days of 12 h/12 h light/dark cycle culturing, sand on the root surface was gently washed with water. Sixteen seedlings were collected, and the water on the root surface was dried with filter paper. The fresh weight of the roots was measured using a 1/10000 balance. Photographs were taken after the roots were separated as far as possible from each other under a Nikon SMZ800 microscope, as shown in **Supplementary Figure 4**.

### 2.3 Nematode acquisition and inoculation

*M. incognita* was maintained on the tomato cultivar L402 in a greenhouse. Tomato plants were gently removed from the pots under a running tap. Egg masses were picked from the root surface using tweezers and placed in Petri dishes with distilled water. The collected egg masses were surface-sterilized with 0.5% sodium hypochlorite for 5 min, rinsed with sterile water, and hatched in the dark at 28°C. Freshly hatched second-stage juveniles (J2s) were collected over two days.

For inoculation assays, the freshly hatched J2s concentration was adjusted in 1% sodium carboxymethylcellulose to approximately 1000 ml^-1^, and seedlings were drenching inoculated with 1 ml of the J2s suspension per plant at the four-leaf stage. Sterile water was used for the control group. The roots of *A. thaliana* seedlings were collected after gently washing the residual sand on their surface.

### 2.4 RNA isolation and quantitative reverse transcription-PCR (RT-qPCR)

For each replicate, total RNA from *Arabidopsis* roots was isolated from three plants in each experiment, and the experiment was independently performed twice. Total RNA was extracted using TRIZOL^®^ reagent (ComWin Biosciences, Beijing, China) according to the manufacturer’s instructions. A NanoDrop 2000 UV-Vis spectrophotometer (Thermo Scientific, Waltham, MA, USA) was used to determine the quality of the RNA samples prior to reverse transcription. cDNA was used for reverse transcription with an oligo (dT) primer using the PrimeScript™ RT Reagent Kit with gDNA Eraser (Takara, Tokyo, Japan). RT-qPCR was performed using the CFX Connect real-time PCR system (Bio-Rad, Hercules, CA, USA). The reactions were performed in a total volume of 25 µL using SYBR^®^ Premix Ex Taq™ II (Takara, Tokyo, Japan). All reactions were performed under the following conditions: an initial denaturation step (30 s at 95°C), followed by 40 cycles of denaturation (5 s at 95°C), annealing (34 s at 60°C), and a melting curve reaction from 60°C to 95°C, with an increase of 0.5°C every 5 s. Col-0 plants were used as the controls. Five biological replicates and three technical replicates were used for each sample. *Actin 8* was selected as the reference gene, and relative gene expression levels were calculated according to the method (Livak and Schmittgen, 2002; Livak and Schmittgen, 2013). The primers used for RT-qPCR assays are listed in **Supplementary Table S1**.

### 2.5 Nematode penetration and development experiment

When the fourth leaf of *Arabidopsis* was fully stretched, the nematode suspension was mixed with an equal volume of 1% sodium carboxymethyl cellulose, and each seedling were drenching inoculated with about 1000 J2 and water with an equal volume of 1% sodium carboxymethyl cellulose were used in the control group. *Arabidopsis* roots were collected after gently washed the residual sands on the roots surfaces from 15 seedlings for each treatment at 18 days post inoculation (dpi). The use of the sodium hypochlorite-acid fuchsin staining method to dye the worms in the whole root tissue can aid in the observation of nematode development stages and the quantification of the number of worms of different instars in the roots. *Arabidopsis* root tissues were stained using the method described by Teixeira et al. (2016). At 18 dpi, the number of galls and juveniles (second-stage juveniles, sausage [second-stage juveniles to third-stage juveniles], globose [third-stage juveniles to fourth-stage juveniles]) was counted for each plant under a Nikon SMZ800 stereoscope.

### 2.6 Observation of GUS and YFP signal

GUS experiments were performed on *Arabidopsis* AtSWEET1-GUS infected and uninfected transgenic root tissues that were taken from the soil at 18 dpi. Roots were processed by double staining as described by Teixeira (2017), with modifications. Briefly, seedlings were infiltrated overnight at 37°C in the dark with GUS-staining buffer containing X-Gluc (Real-Times Biotechnology, Beijing, China) for 20 min, and root tissues were then cleaned with 70% ethanol and 50% ethanol. Roots were washed in water and stained with acid fuchsin, as described previously. The expression of GUS in the roots was examined under an Olympus BX 53 microscope, and images were captured using a coupled Olympus DP 80 digital camera (Tokyo, Japan).

The *A. thaliana* complemented lines (AtSWEET1-YFP-w5-2 and AtSWEET 1-YFP-S2-2) were inoculated with *M. incognita* as described above. Roots were collected at 18 dpi for microscopybasd examination. Arabidopsis roots were cut into small sections, placed on a glass slide, covered with a coverslip after a water droplet was added, and observed microscopically. Observations were carried out using a Olympus FV3000 laser scanning confocal microscope (Tokyo, Japan) with an excitation and emission wavelength of 488 and 513 nm, respectively.

### 2.7 Statistical analysis

Statistical analyses were performed using Microsoft Office Excel 2016, and significance analysis was performed using SPSS 22.0. Student’s *t*-test was used for significance analysis between two treatments, and Duncan’s test was used for the analysis of more than three treatments. The error is shown as the standard deviation between the biological replicates. For the collection and mapping of real-time fluorescence quantitative PCR data, GraphPad Prism 7.00 software was utilized.

## 3 Results

### 3.1 AtSWEETs expression patterns in *Arabidopsis* roots after inoculation with *M. incognita*

**Figure 1:**
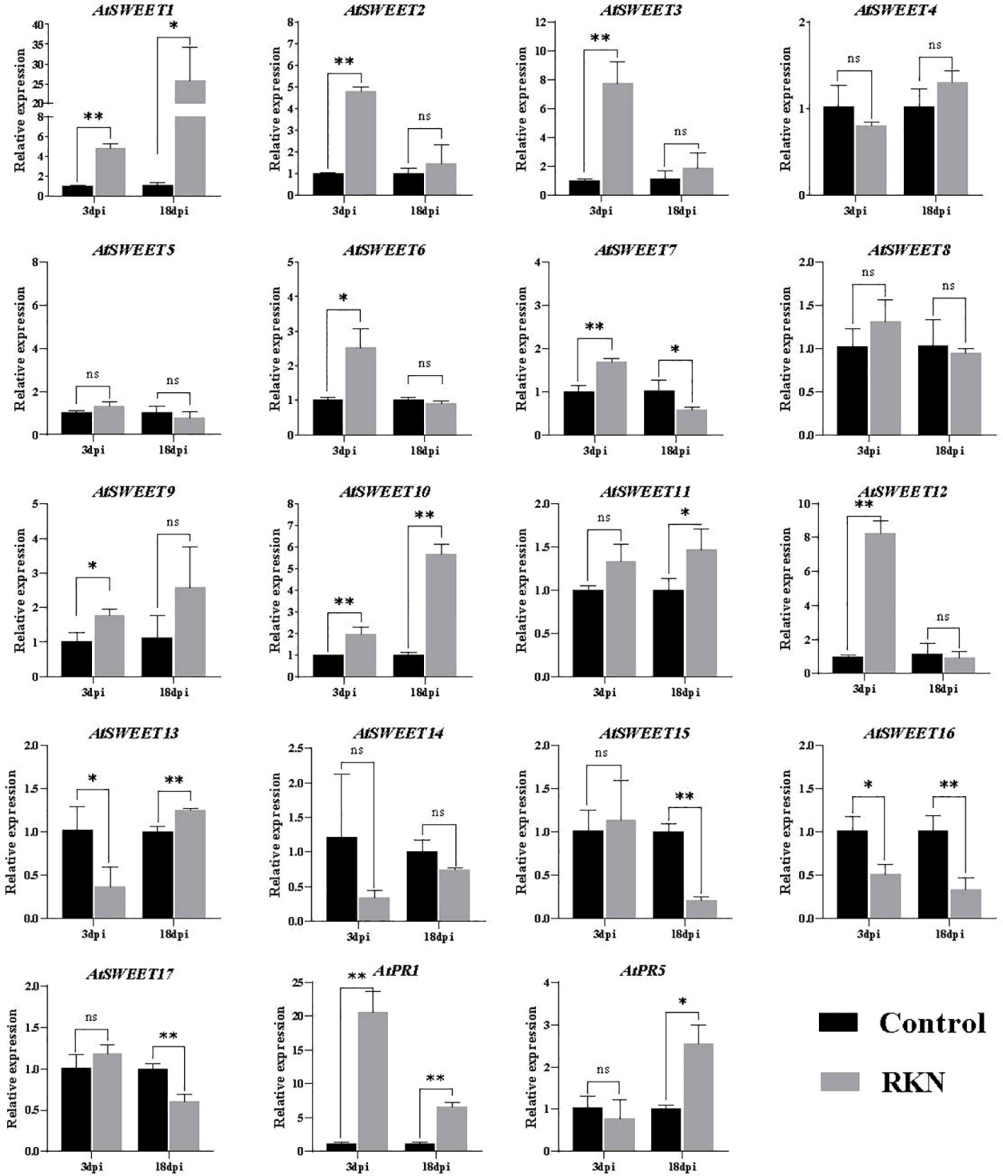
RKNs infection-dependent expression of *AtSWEETs*, *AtPR1*, and *AtPR5* in *A. thaliana* roots. Using noninoculated roots as control, expression levels of *AtSWEETs*, *AtPR1*, and *AtPR5* in *A. thaliana* roots inoculated with *M. incognita* at 3 dpi and 18 dpi were analyzed using quantitative reverse transcription PCR. Five biological replicates and three technical repeats were performed per sample. The *Actin 8* gene was used as an internal control. Error bars indicate the SD between technical repeats (n=3). p-values were analyzed using student’s t-test (p*<0.05; p**<0.01). ns, no significant difference.

*M. incognita* penetrates the roots of *Arabidopsis* and migrates intercellularly, establishing a permanent feeding site. At 2 and 3 dpi, most J2s were observed inside the root tip and vascular cylinder, and feeding sites began to appear at 4 dpi (Teillet et al., 2013). To test which SWEETs respond to the infection, we tested the expression patterns of SWEET family genes by challenging with *M. incognita* at 3 (penetration stage) and 18 (development stage) dpi. The expression of *AtSWEET1*, *AtSWEET2*, *AtSWEET3*, *AtSWEET6*, *AtSWEET7*, *AtSWEET9*, *AtSWEET10*, AtSWEET11, *AtSWEET12*, and *AtSWEET13* in *Arabidopsis* roots were up-regulated by *M. incognita* infection. Among them, *AtSWEET1* was most significantly induced. In contrast, the expression of *AtSWEET7*, *AtSWEET15*, *AtSWEET16*, and *AtSWEET17* was down-regulated at 18 dpi (**Figure 1**). Pathogen-related (PR) genes, such as *PR-1* and *PR-5*, were used as marker genes (Uehara et al., 2010; Hamamouch et al., 2011), and *AtPR1* and *AtPR5* expression levels were significantly induced by RKN infections in roots. These data suggest that these AtSWEETs may be involved in RKN infections.

### 3.2 *AtSWEET1* expression is induced specifically at the galls in roots

**Figure 2:**
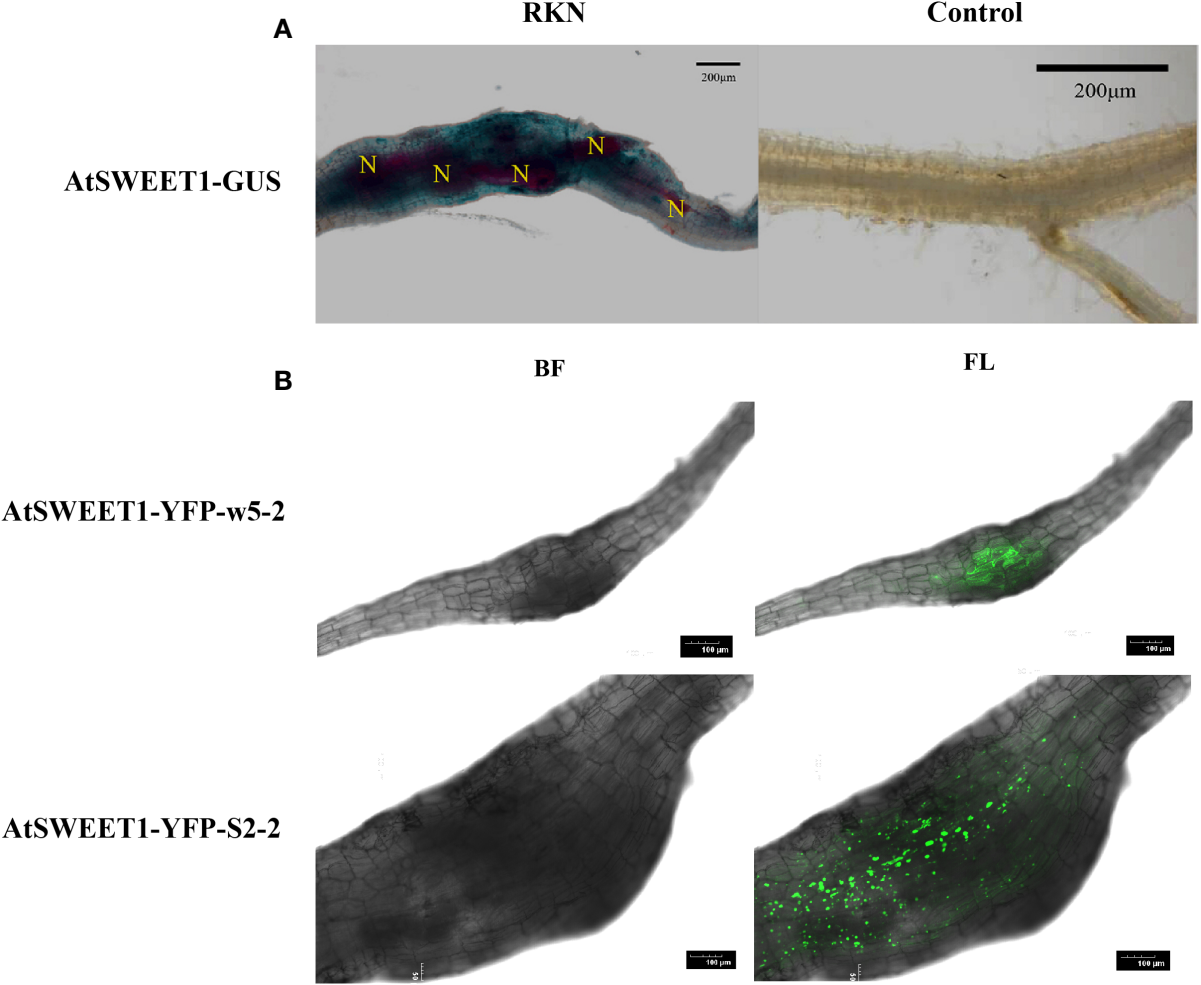
*AtSWEET1* expression is induced specifically at the galls in roots. **(A)** Histochemical GUS assay of AtSWEET1-GUS lines infected with J2 of *M. incognita* at 18 dpi. Strong GUS activity in the nematode feeding sites of AtSWEET1-GUS but no GUS activity in the root of no nematode-infected AtSWEET1-GUS lines was observed. N, nematode. Scale bars=200 μm. **(B)** The LSCM micrographs of *A. thaliana* complemented (AtSWEET1-YFP-w5-2 and AtSWEET1-YFP-S2-2) plants inoculated with *M. incognita*. Scale bars=100 μm.

During the process of *Arabidopsis* inoculation, galls formed on the roots serve as a nutrient sink. *AtSWEET1* was the most responsible *AtSWEET* gene during the infection based on our expression analysis. We investigated whether AtSWEET1 specifically accumulated in galls after infection. Microscopic analysis was performed of GUS-expression patterns upon RKNs infection in Arabidopsis plants expressing AtSWEET1-GUS under the control of the endogenous promoter was investigated (**Figure 2A**). At 18 dpi, a strong GUS signal was observed within the developing gall of *M. incognita* in *Arabidopsis* roots. However, GUS expression was not detectable in uninfected regions of roots. Additionally, AtSWEET1-YFP expressing plants (AtSWEET1-YFP-w5-2 and AtSWEET1-YFP-S2-2) under the native promoter were examined after *M. incognita* inoculation. Similar to the GUS expression pattern, the YFP signal was specifically observed in the galls of the transgenic plant roots (**Figure 2B**).

### 3.3 The invasion and development of RKNs were delayed in *atsweet1* mutant

**Figure 3:**
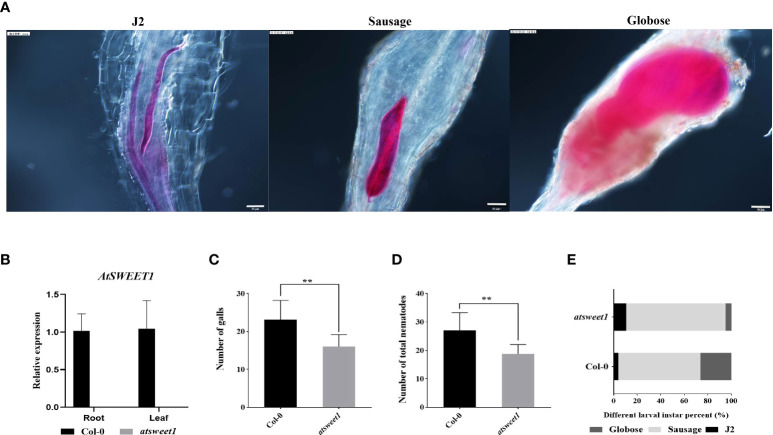
Response of *A. thaliana atsweet1* mutants and wild-type Col-0 plants to *M. incognita* infection. **(A)** The developmental morphology of nematodes at the J2 stage, Sausage stage, and Globose stage. **(B)** The relative expression levels of the *AtSWEET1* gene in *atsweet1* mutant plants were analyzed through quantitative reverse transcription PCR. Using col-0 plants as control, five biological replicates and three technical repeats were performed per sample. The *Actin 8* gene was used as an internal control. **(C)** The number of galls in the root of Arabidopsis inoculated with *M. incognita* at 18 dpi. The number of galls is significantly reduced in the *atsweet1* mutant plants than in the Col-0 plants; **(D)** It shows the total number of nematode-infected *Arabidopsis*. **(E)** Percentage of nematodes corresponding to different developmental stages (J2 stage; Sausage stage; Globose stage) in the *atsweet1* mutants and wild-type Col-0 at 18 dpi (n = 15). Data are presented as mean ± SD, and p-values were analyzed using student’s t-test (p**<0.01). Scale bars=50 μm.

Since *AtSWEET1* was specifically induced in the galls by inoculation with *M. incognita*, the function of *AtSWEET1* in plant-nematode interactions was investigated. The infestation and development of *M. incognita* were examined using the sodium hypochlorite-acid fuchsin staining method at 18 dpi in *Atsweet1* mutants, complemented (*AtSWEET1*-YFP-w5-2 and *AtSWEET1*-YFP-S2-2), and wild-type Col-0 plants. The reference morphology of nematodes at different developmental stages is shown in **Figure 3A**. RT-qPCR results showed that no transcript was detected in the atsweet1 mutant compared to the wild-type Col-0 plants (**Figure 3B**). Inoculation with *M. incognita* revealed that the number of galls and the total nematode population in *atsweet1* mutant plants was reduced when compared to the Col-0 plants (**Figures 3C, D**), which indicates that AtSWEET1 negatively regulates plant susceptibility to root-knot nematode disease. At 18 dpi, the percentage of nematodes at the J2 stage in *atsweet1* mutant roots was substantially higher than that in the Col-0 plants, while the proportion of nematodes at the sausage and globose stages was significantly lower in the *atsweet1* mutant roots than in Col-0 plants (**Figure 3E**).

**Figure 4:**
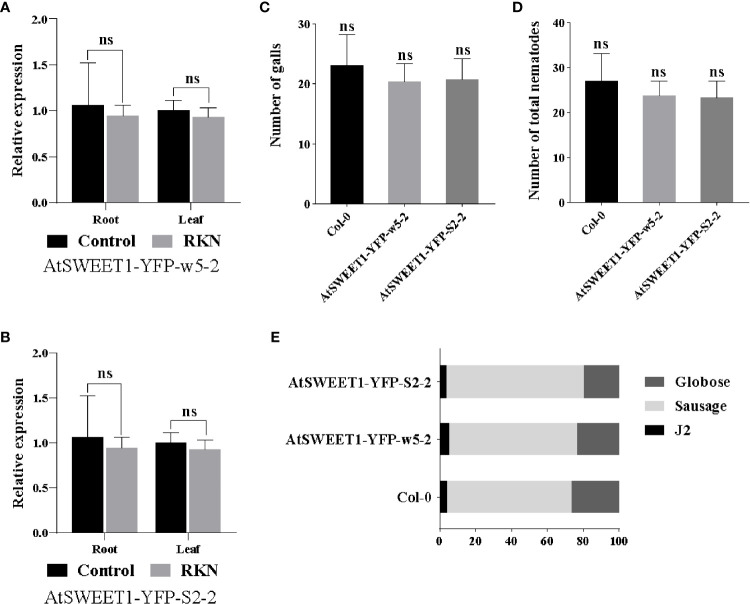
Response of *A. thaliana* complemented (AtSWEET1-YFP-w5-2 and AtSWEET1-YFP-S2-2) and wild-type Col-0 plants to *M. incognita* infection. **(A, B)** The relative expression levels of the *AtSWEET1* gene in *A.thaliana* complemented plants were analyzed using quantitative reverse transcription PCR. Using col-0 plants as control, five biological replicates and three technical repeats were performed per sample. The *Actin 8* gene was used as an internal control. **(C)** The number of galls in the root of Arabidopsis inoculated with *M. incognita* at 18 dpi. There is no significant difference between complemented plants and Col-0 plants. **(D)** The total number of nematode-infected *Arabidopsis*. **(E)** Percentage of nematodes corresponding to different developmental stages (J2 stage; Sausage stage; Globose stage) in the complemented plants and Col-0 plants at 18 dpi (n = 15). Data are presented as mean ± SD, and p-values were analyzed using student’s t-test. ns, no significant difference.

To verify whether the *atsweet1* phenotype was caused by the loss of function of *AtSWEET1*, AtSWEET1-YFP was expressed under the control of the *pAtSWEET1* promoter. RT-qPCR showed that *AtSWEET1* expression levels were similar between Col-0 and the complementation lines (AtSWEET1-YFP-w5-2 and AtSWEET1-YFP-S2-2) (**Figures 4A, B**). Inoculation with *M. incognita* revealed that the number of galls and the total nematode populations were not significantly different between complementation and Col-0 plants (**Figures 4C, D**), nor were there differences in the proportion of different nematode stages between complementation and Col-0 plants (**Figure 4E**).

### 3.4 Sucrose transporters AtSWEET10, 13, 14 have a potential role in *M. incognita* infection

**Figure 5:**
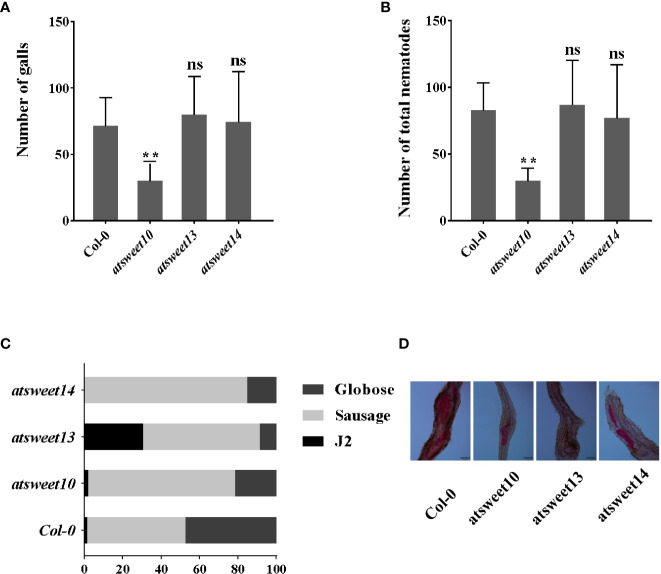
Response of *A. thaliana* mutants and wild-type Col-0 plants to *M. incognita* infection. **(A)** The number of galls in the root of *Arabidopsis* inoculated with *M. incognita* at 18 dpi. **(B)** The total number of nematode-infected *Arabidopsis*. **(C)** Percentage of nematodes corresponding to different developmental stages (J2 stage; Sausage stage; Globose stage) in the mutants and wild-type Col-0 at 18 dpi (n = 15). **(D)** The major developmental morphology of nematodes in Col-0, *atsweet10*, *atsweet13*, and *atsweet14* mutants at 18 dpi. Data are presented as mean ± SD, and p-values were analyzed using student’s t-test (p**<0.01). ns, no significant difference. Scale bars=100 μm.

Chen et al. (2010) reported that *AtSWEET10* expression is highly induced by *Pseudomonas syringae* pv. *tomato* strain DC3000, and the expression of *AtSWEET13* was induced by *G. cichoracearum* and *B. cinerea*. *AtSWEET13* and *AtSWEET14* may be involved in modulating GA response in *Arabidopsis* (Yuri et al., 2016). The expression patterns showed that sucrose transporters AtSWEET10, AtSWEET13, and AtSWEET14 were induced by *M. incognita* inoculation. Using the sodium hypochlorite-acid fuchsin staining method, infestation and development of *M. incognita* in *Arabidopsis* roots were investigated at 18 dpi in *atsweet10, atsweet13*, and *atsweet14* mutants and wild-type Col-0 plants (**Figure 5**). The galls and total nematodes were reduced in *atsweet10* mutant roots compared to Col-0 plants (**Figures 5A, B**), but no significant changes were observed between Col-0, *atsweet13*, and *atsweet14* roots (**Figures 5A, B**). In addition, the development of nematodes was hampered in each mutant plant (**Figures 5C, D**).

## 4 Discussion

The plant genome contains three major sugar transporters: the sucrose transporter (SUT/SUC), sugar transporter (STP), and SWEETs (Doidy et al., 2012; Chen et al., 2015). SWEETs aid the pathogen’s prey on the host’s nutrients during the infection process. Among the 21 rice SWEET gene family members, five (*OsSWEET11-15*) have been shown to provide nutrition to *Xanthomonas oryzae* pv. *oryzae* (Chu et al., 2006; Yang et al., 2006; Yuan et al., 2009; Römer et al., 2010; Liu et al., 2011; Yu et al., 2011; Li et al., 2013; Streubel et al., 2013; Zhou et al., 2015). When the *VvSWEET4* gene in grapes was inoculated with *Pythium teroegenes*, the glucose content in the hairy roots increased, and the expression was significantly induced (Meteier et al., 2019). The TAL20 effector of bacterial blight (*Xanthomonas axonopodis* pv. *manihotis*) can cause high levels of *MeSWEET10a* expression in cassava (*Manihot esculenta*), allowing sucrose in the mesophyll cells to be transported outside the cell, where pathogenic bacteria can easily use it to increase infectivity (Cohn et al., 2014). With respect to plant parasitic nematodes, several SWEET genes are triggered and expressed by nematode infections, and they also play an important role in the interaction between nematodes and host plants (Redding et al., 2018; Zhao et al., 2018). In this study, the mRNA levels of *Arabidopsis AtSWEET1*, *AtSWEET2*, *AtSWEET3*, *AtSWEET6*, *AtSWEET7*, AtSWEET9, *AtSWEET10*, *AtSWEET11*, *AtSWEET12*, and *AtSWEET13* were highly upregulated during *M. incognita* infection.

*M. incognita*, a root-knot nematode (RKN), severely threatens plant growth and yield. RKNs infect the roots and trigger the formation of giant cells. Giant cells are the sink tissue where RKNs hijack nutrients from host plants (Hammes et al., 2005; Caillaud et al., 2008). Previous research has found that the metabolism of sugars is accelerated in galls generated following inoculation with *M. incognita*, and the contents of starch, sucrose, and glucose showed obvious accumulation (Baldacci-Cresp et al., 2012). As a result, the metabolism and transport of sugars play an important role in the parasitic process of *M. incognita*. *AtSUC2* and *AtSUC4* are activated by *H. schachtii* and mediate the transmembrane transport of sucrose in the syncytia (Juergensen et al., 2003). The significant upregulation of *AtSUC4* in galls after inoculation with *M. incognita* suggests its sucrose-supplying role in *Arabidopsis* (Hofmann et al., 2009). Transcriptome analyses of wheat interaction with *Heterodera avenae* revealed that the bidirectional sugar transporter SWEET12 was significantly induced at 3 dpi, and the genes encoding sugar transport protein 4 and sugar carrier protein A were upregulated at 8 dpi (Qiao et al., 2019). The relative expression of 50 transporter genes from 18 different gene families was significantly changed in response to RKN infections in *Arabidopsis*, such as *AtSUC1*, *AtSTPs*, and *AtSFPs* (Hammes et al., 2005). Although the role of sugar transporters in the interaction with root-knot nematodes is poorly understood, it is plausible that sugar transporters are expressed in giant cells, and that sugars might be imported into giant cells *via* sugar transporters (Hofmann et al., 2009; Bartlem et al., 2014).

Giant cells are extremely metabolically active, and high levels of total protein, amino acids, glucose, glucose 6-phosphate, and ATP are found in RKN-induced giant cells (Gommers and Dropkin, 1977). The sucrose transporter gene *AtSUC1* was significantly induced upon RKN infestation. The expression of this gene is also higher in the feeding sites than in the surrounding tissue (Hammes et al., 2005). It was recently reported that plant-specific membrane trafficking mechanisms might be involved in gall formation (Suzuki et al., 2021). We examined the status of reactive oxygen species, callose, and PTI-related genes in the roots of the *atsweet1* mutant and Col-0 inoculated with RKNs, and found no significant differences (**Supplementary Figures 1**–**3**). To examine whether *AtSWEETs* influence the parasitism of *M. incognita* on *A. thaliana*, *atsweet1*, *atsweet10*, *atsweet13*, and *atsweet14* mutant plants were inoculated with RKNs. Nematode infection assays were performed with significantly lower numbers of galls and slower development of juveniles in *atsweet1* and *atsweet10* mutant roots than in wild-type plants. AtSWEET1 and AtSWEET10 transport different types of sugars and may have different functions during RKN infections.

In *Arabidopsis*, there is a significant difference in the expression of sugar transporter genes (Hammes et al., 2005; Zhao et al., 2018) and genes related to other physiological activities (Suzuki et al., 2021) in plants inoculated with parasitic nematodes. Sugar transporter genes *AtSUC2* and gall specific promoter *TobRB7*, have been identified to be involved in the feeding site of nematodes (Opperman et al., 1994; Juergensen et al., 2003). Recently, sucrose transport was found to be mostly dependent on plasmodesmata-mediated sucrose supply from the rice root phloem to *M. graminicola*-caused giant cells, and *OsSWEET11 to 15* and *OsSUTs* play no major role in this process (Xu et al., 2021). In our experimental results, GUS activity in the *AtSWEET1* reporter was not observed in non-galls of the root, which is consistent with Chen et al. (2010) finding that the *AtSWEET1* gene is not expressed in the root but *M. incognita*-caused galls of AtSWEET1-GUS lines (**Figure 2A**). Similarly, we observed YFP fluorescence intensity in galls formed by RKN infection (**Figure 2B**). Meanwhile, the RT-qPCR assays showed that the *AtSWEET1* gene was significantly upregulated at 18 dpi (**Figure 1**). These results indicate that the expression of *AtSWEET1* near galls in *Arabidopsis* roots is induced by *M. incognita*.

AtSWEET1-GUS or AtSWEET1-YFP was localized in the galls, implying a potential function of AtSWEET1 in plant-RKN interactions. Inoculation of RKNs revealed that *atsweet1* mutants inhibited parasitism of RKNs, with a reduced total RKN number and inhibition of RKN development. Expression of AtSWEET1-YFP in *atsweet1* successfully restored the number and development of RKNs to the wild-type level, suggesting that AtSWEET1 indeed negatively regulates plant defense against RKNs. This might be due to the modulation of sugar availability in the giant cells inside the galls. In addition, the results suggest that the YFP function at the C-terminus of AtSWEET1 did not affect its function, which further confirmed that AtSWEET1-YFP localization at the galls is confident. In this study, we mainly discuss the fructose transporter AtSWEET1. The number of galls, the total populations of nematodes, and the proportion of nematodes at different stages were not significantly different between complemented and Col-0 plants (**Figures 4C, D**), but patterns were completely reversed in *atsweet1* mutant roots (**Figure 3**).

In the phloem sugar-loading process, SWEETs and SUTs controlled the last two steps. AtSWEET11 and AtSWEET12 efflux sucrose from the phloem parenchyma cells to the apoplast for SUT import into companion cells for long-distance transport (Chen et al., 2012). AtSUC4 was expressed in galls, implying that sugar loading in galls might also require SWEET and SUT members. In this study, *AtSWEET10* was induced by RKN inoculation, and its mutant inhibited RKN parasitism, suggesting that AtSWEET10 may play an important role in sucrose transport before it is transported into giant cells *via* SUC4. STP is an importer that is activated by infection with *Pseudomonas syringe* and transports apoplastic glucose into cells to reduce the sugar content in the apoplast (Bezrutczyk et al., 2018). As AtSWEET1 is a uniporter and effluxes glucose, it might also be possible that AtSWEET1 effluxes sugars in neighboring cells of giant cells, and STP will import glucose into giant cells, similar to the SWEET-SUT module. AtSWEET1 is a member of clade I, which are glucose transporters, whereas AtSWEET10, AtSWEET13, and AtSWEET14 are members of clade III, which serve as sucrose transporters (Chen et al., 2010). We assessed the changes of total sugar, glucose, fructose, galactose, and sucrose content in *atsweet1* and Col-0 roots. These results do not completely match our previous results (**Supplementary Figure 5**). The reason for this might be that we used whole roots instead of galls, that the sugars in the nematodes in the roots could not be excluded in the detection process; or *AtSWEET11* and *AtSWEET12*, which are involved in phloem transport in Arabidopsis after *AtSWEET1* mutation, might also be involved in nematode infection. Relative expression of *AtSWEET12* gene was induced by RKNs infection (**Figure 1**). We hypothesized that in addition to AtSWEET1 regulating sugar transport across the cells near the AtSWEET12, which engages in phloem transport, affects the change of sugar content in the roots of *atsweet1* mutants inoculated with RKNs.

In this paper, we discuss the roles of AtSWEET1 in *A. thaliana* infected by *M. incognita*. The assays on the changes of total sugar in roots of *Arabidopsis* after inoculated with RKNs showed that not only AtSWEET1 played a role. Meanwhile, qPCR assays showed that the expression of *AtSWEET12* gene was induced by RKNs, and the development of nematodes in *atsweet10* mutant was impact. By integrating the results of all assays, we can conclude that there are multiple SWEET sugar transporters involved in the interaction between *Arabidopsis thaliana* and RKN and *AtSWEET1* gene negatively regulates plant defenses to root-knot nematode disease.

## Data availability statement

The original contributions presented in the study are included in the article/**Supplementary Material**. Further inquiries can be directed to the corresponding author.

## Author contributions

XZ, YX, YZ, and DZ conceived and designed the experiments. YZ and DZ performed experiments. XZ, YZ, and DZ analyzed the data. L-QC, YX, YD, LC, HF, YW, and XL contributed to the analyses and provided materials and reagents. XZ, YX, and YZ wrote the manuscript. All authors contributed to the manuscript and approved the submitted version.
