# Supplementary material for: AtSWEET1 negatively regulates plant susceptibility to root-knot nematode disease
Source: Front Plant Sci. 2023 Feb 7;14:1010348. doi: 10.3389/fpls.2023.1010348 (PMC9941640; doi:10.3389/fpls.2023.1010348)
Supplement: Supplementary file 1 [file DataSheet_1.docx]

Supplementary Material

# Supplementary Data

## Observation of Hydrogen Peroxide

The *Arabidopsis Thaliana* Col-0 and *atsweet1* mutant seeds were planted on the sands surface. About 1000 larvae of the second stage of RKNs were inoculated in roots. At 18 days post inoculation (dpi), the sands on the root surface were washed gently with water, and reactive oxygen species staining was performed with the plant hydrogen peroxide staining solution (DAB method) kit (Servicebio, Hubei, China). Roots were put into DAB staining solution at room temperature and protected from light for 4 hours. After taking them out, they were rinsed five times in pure water. After being sucked dry, they were put into DAB preservation solution for 30 minutes. Olympus BX 53 microscope was used for observation, and images were obtained with Olympus DP 80 digital camera (Tokyo, Japan).

## Observation of Callose Deposition

At 18 days post inoculation (dpi), the sands on the root surface were washed gently with water, and fresh roots were directly immersed in Carnoy fixative solution (Solarbio, Beijing, China) and fixed for 24 hours. Put in absolute ethanol twice for 1 minute each time, then transfer to at least 10 times the volume of 100% ethanol. Before staining, the roots were immersed in 50% ethanol for 30min, and slightly drained. Then soak in 1×PBS and equilibrate for 30min. Soak in callose staining solution (Solarbio, Beijing, China) and stain for 1 hour at room temperature. The images were taken with an Olympus FV3000 laser scanning confocal microscope (Tokyo, Japan) with an excitation wavelength of 400nm and an emission wavelength of 500nm.

## Expression of PTI Marker Genes

Transcription factor (TF) MYB51, which regulates glucosinolate biosynthesis, as well as TF WRKY11 involved in basal defense (Millet et al., 2010). Root-knot nematode infection induced expression of genes involved *MYB51* and *WRKY11* (Teixeira et al., 2016). Using noninoculated roots as control, expression levels of *AtMYB51*, and *AtWRKY11* in *Arabidopsis* roots inoculated with RKNs at 1 dpi were analyzed using quantitative reverse transcription PCR. Five biological replicates and three technical repeats were performed per sample. Primers used in RT-qPCR of *AtMYB51* (F:5’- ACAAATGGTCTGCTATAGCT-3’; R:5’- CTTGTGTGTAACTGGATCAA-3’) and *AtWRKY11* (F:5’- CCACCGTCTAGTGTAACACTCGAT-3’; R:5’- TGCAACGGAGCAGAAGCAAGGAA-3’).The *Actin 8* gene was used as an internal control.

## Morphology and Fresh Weight of *atsweet1* and Col-0

Taken 16 seedlings that have been growing in sands for 35 days, gently wash the sands on the root surface with water, blot the water on the root surface with filter paper, and weigh the mass with 1/10000 electronic balance. Results indicated that there was no difference in root fresh weight between *Arabidopsis* Col-0 and *atsweet1*.

## The Sugar Content in the Root

Considering the function of SWEET as a sugar transporter, additional tests to measure changes in plant sugar content are highly acceptable. We added assays compare the content of glucose in roots between the *atsweet1* mutant and WT lines. 30 roots have been used to test the sugar content through high-performance liquid chromatography and we have repeated the assays twice. We expected root sugar content to be reduced in *atsweet1* mutants compared with wild-type, and even less after inoculation with RKNs. But according to the results, we cannot explain the phenotype. We thought that the reason might be that we used whole roots instead of galls, that the sugars in the nematodes in the roots could not be excluded in the detection process, and that AtSWEET11 and AtSWEET12, which are involved in phloem transport in *Arabidopsis* after *AtSWEET1* mutation, might also be involved in nematodes infection. The relative expression of AtSWEET12 gene was induced by RKNs infection (Figure 1). If laser cutting and other means can be used to cut root knots, it will be more convincing to detect the sugar content in root knots and non-root knots as well as roots without RKNs inoculation. Or some techniques can be used to detect the direction of sugar flow across syncytial cell membrane and the types of sugars flowing in or out of syncytial cells. This will provide more powerful evidence to elucidating the role of sugars and the role of sugar transporters in *Meloidogyne incognita* parasitism.

# Supplementary Figures and Tables

## Supplementary Figures


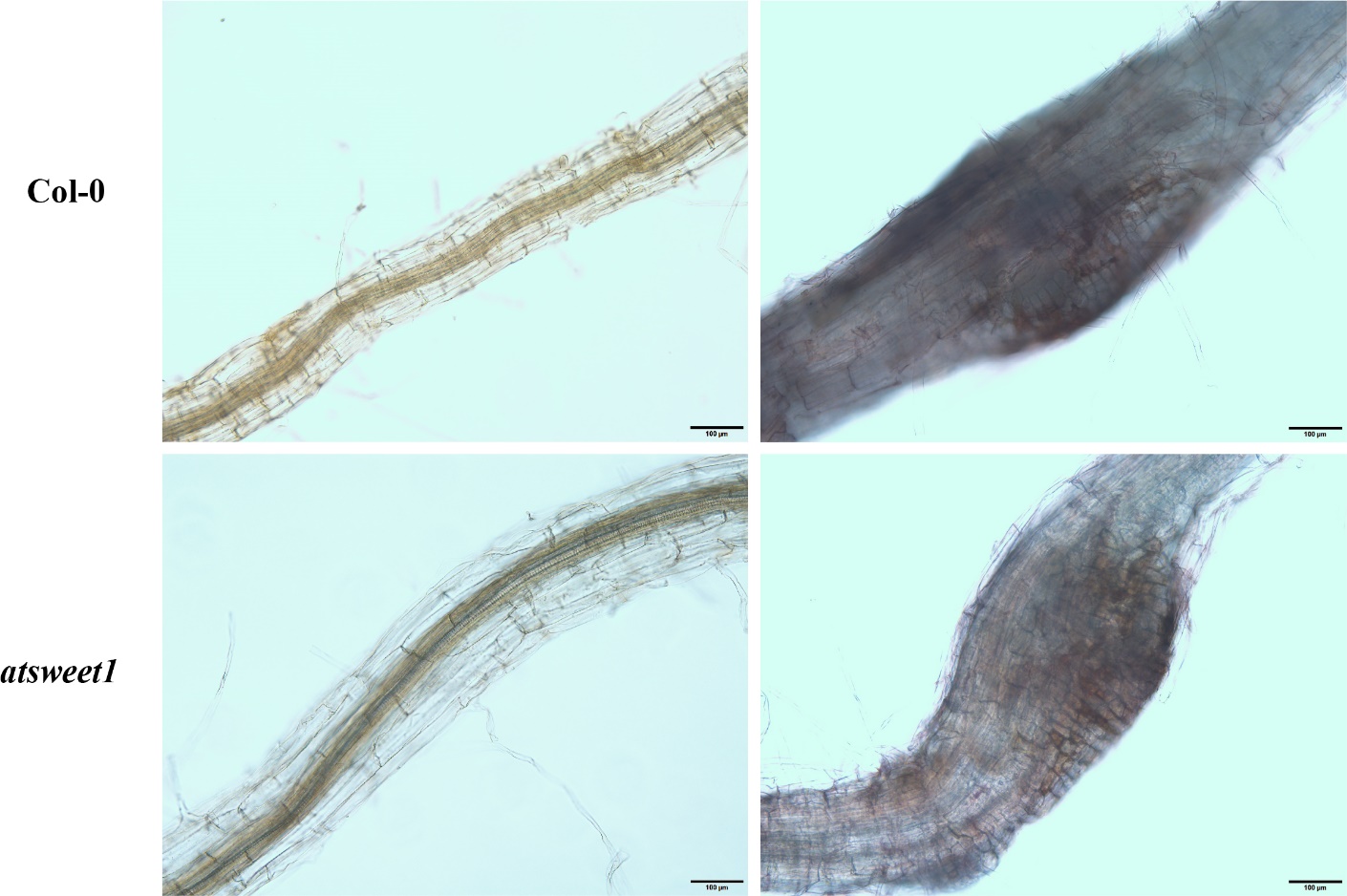


**Supplementary Figure 1.** DAB staining of roots 18 days post inoculation with RKNs. No reactive oxygen species burst was observed where the nematode has not penetrated. The H_2_O_2_ burst occurred only at the site where the nematode penetrated the root. In the roots of Col-0 and *atsweet1* plants, there was no differences of hydrogen peroxide produce. Scale bars=100 μm.


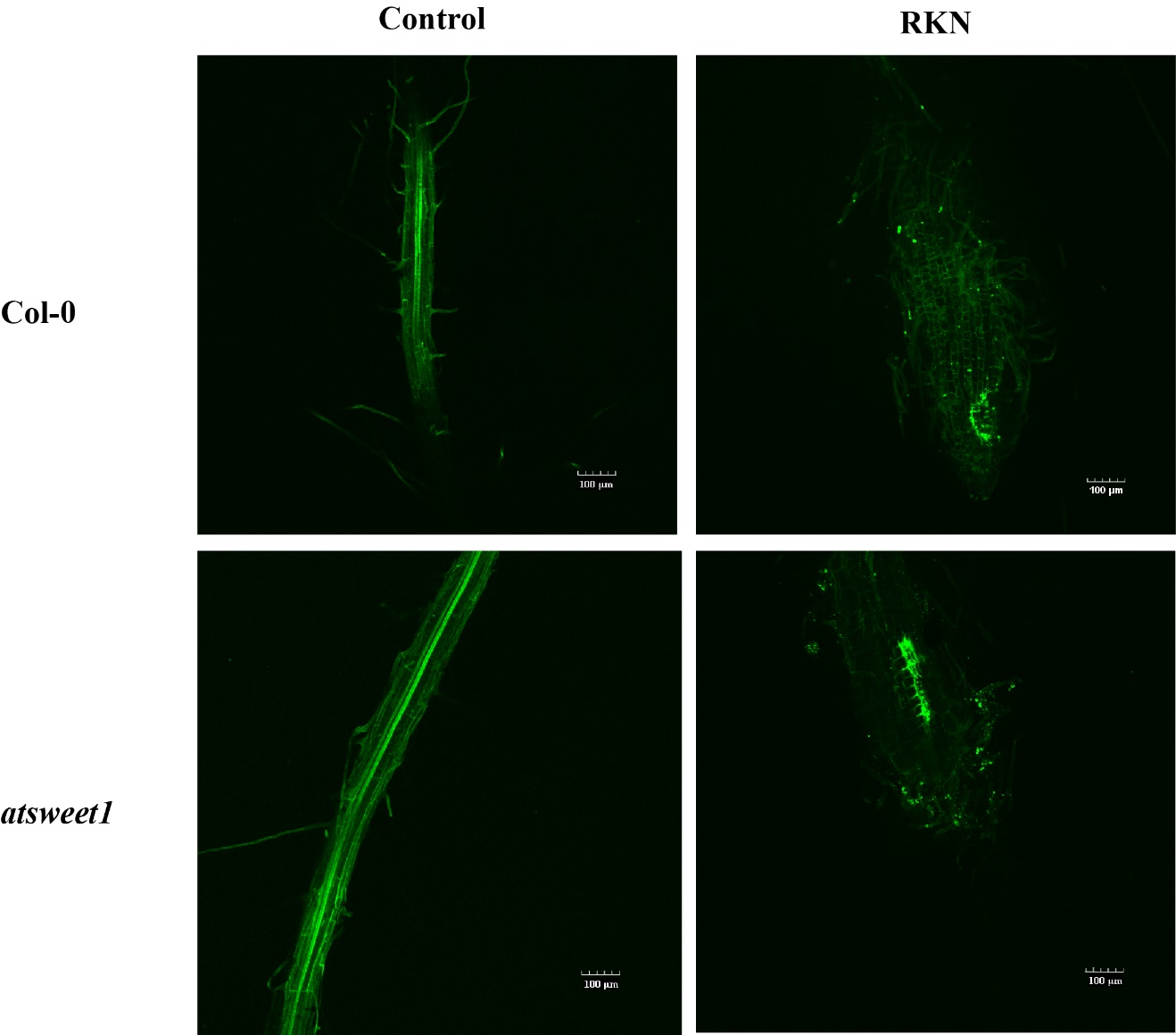


**Supplementary Figure 2.** Callose deposition in the roots. In the roots of Col-0 and *atsweet1* plants, there was no significant differences in callose deposition between Col-0 and *atsweet1* roots inoculated with RKNs at 18 dpi. Scale bars=100 μm.


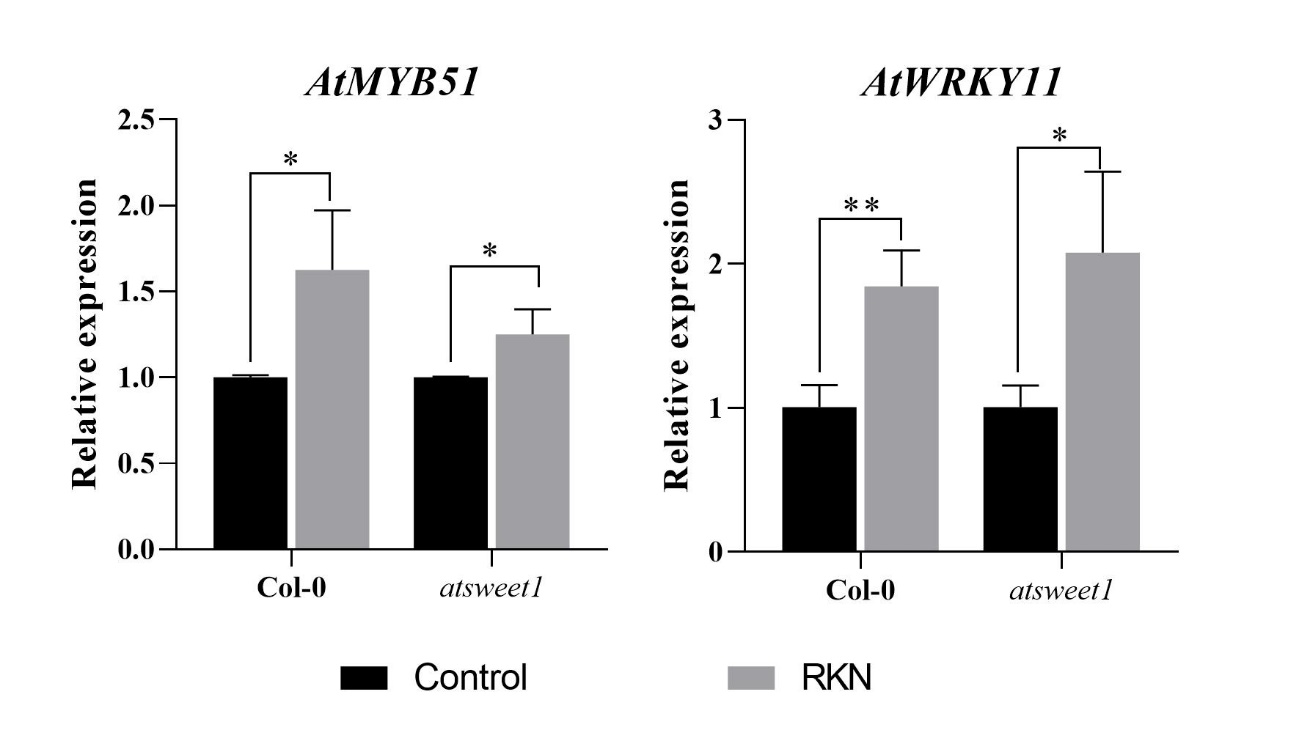


**Supplementary Figure 3.** RKNs infection-dependent expression of PTI marker genes in *A. thaliana* roots. Using non-inoculated roots as control, expression levels of *AtMYB51* and *AtWRKY11* in *A. thaliana* roots inoculated with *M. incognita* at 1 dpi were analyzed using quantitative reverse transcription PCR. Five biological replicates and three technical repeats were performed per sample. The *Actin 8* gene was used as an internal control. Error bars indicate the SD between technical repeats (n=3). p-values were analyzed using student’s t-test (p*<0.05; p**<0.01). ns, no significant difference.


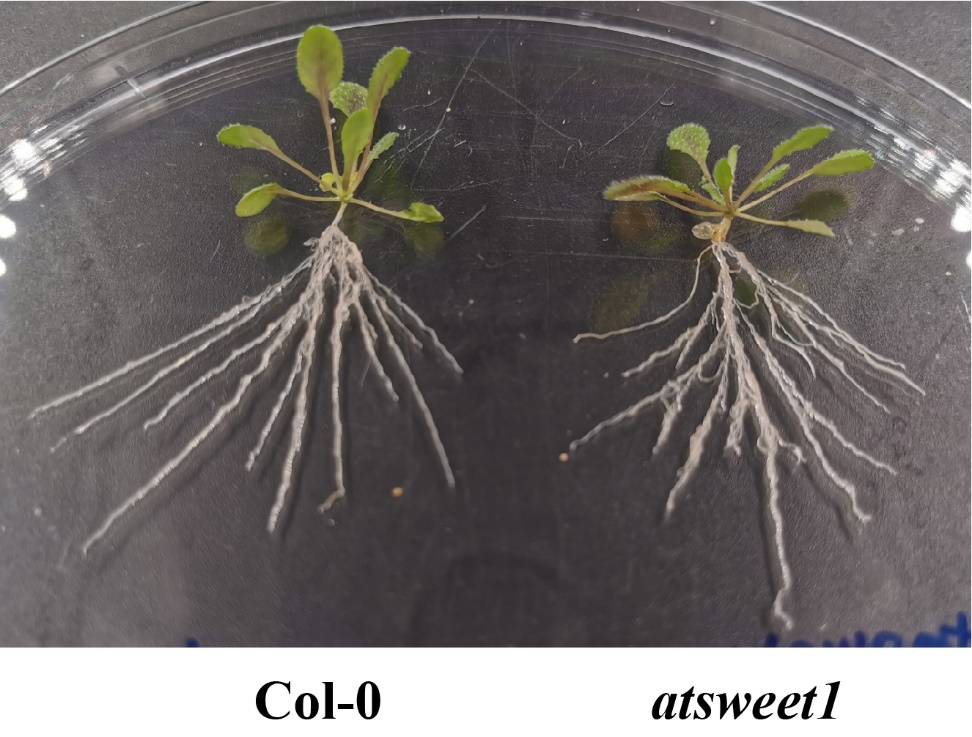


**Supplementary Figure 4.** Morphology of *atsweet1* mutant line and WT line.


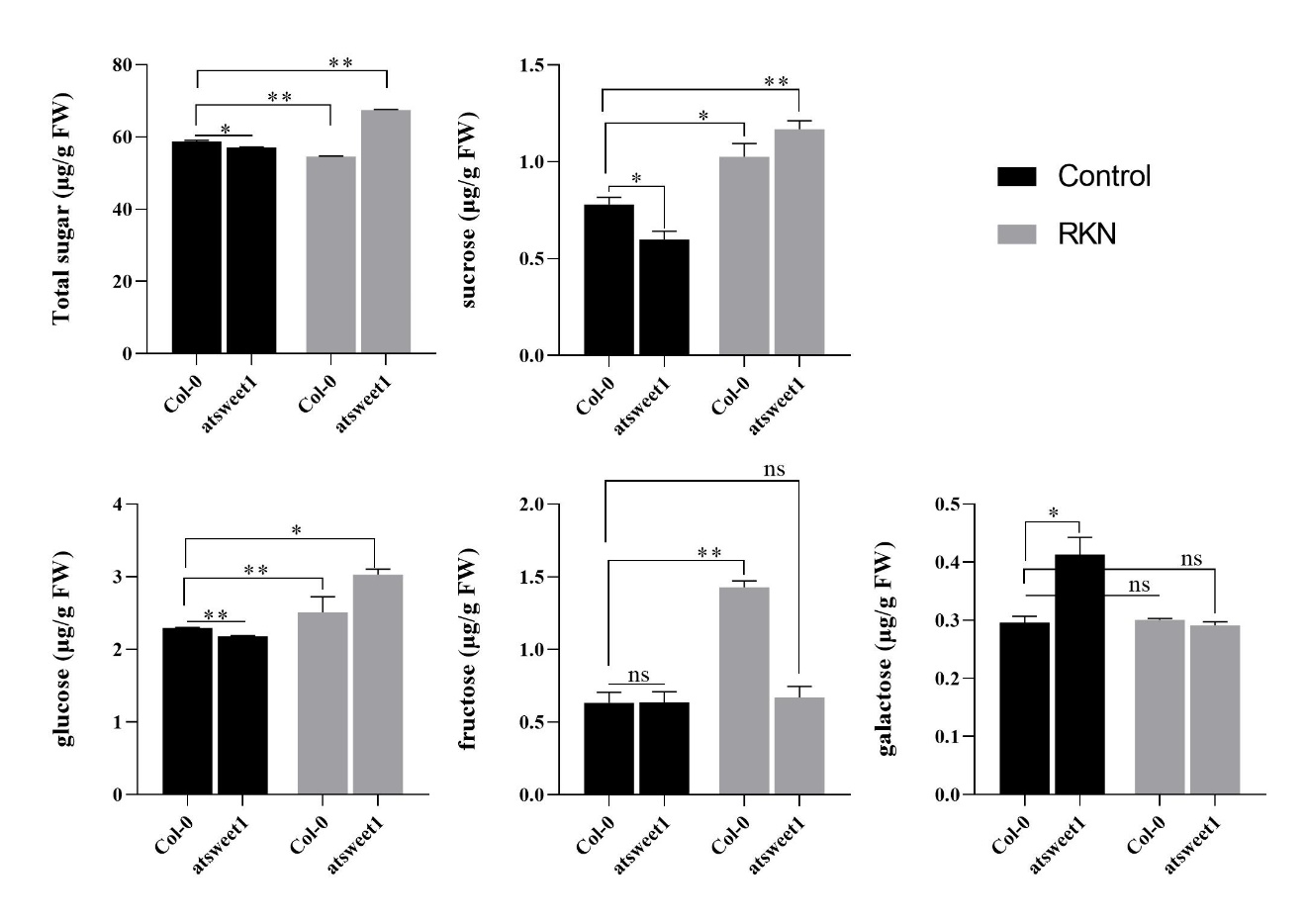


**Supplementary Figure 5.** The sugar content in the root. The content of total sugar, glucose, fructose, galactose and sucrose in Col-0 and atsweet1 mutant roots. Values are means ± SE. **, P < 0.01. *, P < 0.05. ns, no significant difference.

## Supplementary Tables

Table S1. Primers used in this study.

| Genes | Primers | Sequence | purposes |
| --- | --- | --- | --- |
| *AtSWEET1* | AtSWEET1-F | CTTCTCCACTCTCCATCATGAGATT | RT-qPCR |
|  | AtSWEET1-R | CATCTGCAGATTTCTCTCCTTTGT | RT-qPCR |
| *AtSWEET2* | AtSWEET2-F | AACAGAGAGTTTAAGACAGAGAGAAG | RT-qPCR |
|  | AtSWEET2-R | ATCCTCCTAAACGTTGGCATTGGT | RT-qPCR |
| *AtSWEET3* | AtSWEET3-F | CCAACTTTTCCCTAATCTTTGTTCTTC | RT-qPCR |
|  | AtSWEET3-R | AACACCCTTGAAAATGTTACTATTGGA | RT-qPCR |
| *AtSWEET4* | AtSWEET4-F | CCATCATGAGTAAGGTGATCAAGA | RT-qPCR |
|  | AtSWEET4-R | CAAAATGAAAAGGTCGAACTTAATAAGTG | RT-qPCR |
| *AtSWEET5* | AtSWEET5-F | TGACCCTTATATTTTGATTCCAAATGGT | RT-qPCR |
|  | AtSWEET5-R | GCCAAGTTCGATTCCAGCATTC | RT-qPCR |
| *AtSWEET6* | AtSWEET6-F | GACTCGGTTACGTTGGTGAAGT | RT-qPCR |
|  | AtSWEET6-R | CAAACGCCGCTAACTCTTTTGTTTAA | RT-qPCR |
| *AtSWEET7* | AtSWEET7-F | GACCCATTCATGGCTATACCAAAT | RT-qPCR |
|  | AtSWEET7-R | ATCCCATAATCCGAAGTTTAATAACACT | RT-qPCR |
| *AtSWEET8* | AtSWEET8-F | TTGCTCTCTTCTTCATCAATCTCTCT | RT-qPCR |
|  | AtSWEET8-R | AGATCCTCCAGAAAGTCTTCGCT | RT-qPCR |
| *AtSWEET9* | AtSWEET9-F | GCAAGAGAAAGAGAGAAAAGTGAAGA | RT-qPCR |
|  | AtSWEET9-R | CCCATAAAACGTTGGCACTGGT | RT-qPCR |
| *AtSWEET10* | AtSWEET10-F | TAGAGGAAGAGAGAGGGAGAGAGT | RT-qPCR |
|  | AtSWEET10-R | ATATACGAACGAACGTCGGTATTG | RT-qPCR |
| *AtSWEET11* | AtSWEET11-F | TCCTTCTCCTAACAACTTATATACCATG | RT-qPCR |
|  | AtSWEET11-R | TCCTATAGAACGTTGGCACAGGA | RT-qPCR |
| *AtSWEET12* | AtSWEET12-F | AAAGCTGATATCTTTCTTACTACTTCGAA | RT-qPCR |
|  | AtSWEET12-R | CTTACAAATCCTATAGAACGTTGGCAC | RT-qPCR |
| *AtSWEET13* | AtSWEET13-F | CTTCTACGTTGCCCTTCCAAATG | RT-qPCR |
|  | AtSWEET13-R | CTTTGTTTCTGGACATCCTTGTTGA | RT-qPCR |
| *AtSWEET14* | AtSWEET14-F | ACTTCTACGTTGCGCTTCCAAATA | RT-qPCR |
|  | AtSWEET14-R | CAGTTCAACATTAAAGTCAATCACTAATTC | RT-qPCR |
| *AtSWEET15* | AtSWEET15-F | CAATGACATATGCATAGCGATTCCAA | RT-qPCR |
|  | AtSWEET15-R | GGACTCATCACGACAATACTCTTAAG | RT-qPCR |
| *AtSWEET16* | AtSWEET16-F | GAGATGCAAACTCGCGTTCTAGT | RT-qPCR |
|  | AtSWEET16-R | GCACACTTCTCGTCGTCACA | RT-qPCR |
| *AtSWEET17* | AtSWEET17-F | AGTGACAACAAAGAGCGTGAAATAC | RT-qPCR |
|  | AtSWEET17-R | ACTTAAACCGTTGCTTAAACCAACC | RT-qPCR |
| *Actin 8* | ACTIN8-F | CACTTTCCAGCAGATGTGGATC | RT-qPCR |
|  | ACTIN8-R | AATGCCTGGACCTGCTTCAT | RT-qPCR |
| *AtPR1* | AtPR1-F | TCATGGCTAAGTTTGCTTCC | RT-qPCR |
|  | AtPR1-R | AATACACACGATTTAGCACC | RT-qPCR |
| *AtPR5* | AtPR5-F | GTTCATCACAAGCGGCATT | RT-qPCR |
|  | AtPR5-R | GTCAATTCAAATCCTCCATCG | RT-qPCR |
